# Uso de glucocorticoides sistémicos para el tratamiento del asma grave: Consenso multidisciplinar español

**DOI:** 10.1016/j.opresp.2022.100202

**Published:** 2022-09-06

**Authors:** Javier Domínguez-Ortega, Julio Delgado Romero, Xavier Muñoz Gall, Amparo Marco, Marina Blanco-Aparicio

**Affiliations:** a Departamento de Alergia, Hospital La Paz, Instituto de Investigación IDiPAZ, Madrid, España; b Ciber de enfermedades respiratorias CIBERes, Madrid, España; c UGC Alergología, Hospital Virgen Macarena, Sevilla, España; d Servicio de Neumología, Hospital Vall d’Hebron, Barcelona, España; e Servicio de Endocrinología y Nutrición, Complejo Hospitalario Universitario de Toledo (CHUT), Toledo, España; f Servicio de Neumología. Hospital Universitario de A Coruña, A Coruña, España

**Keywords:** Asma, Glucocorticoides, Consenso, Delphi, Retirada de tratamiento, Efectos adversos, Asthma, Glucocorticoids, Consensus, Delphi, Treatment withdrawal, Side effects

## Abstract

**Antecedentes y objetivo:**

Los glucocorticoides sistémicos (GCS) se han utilizado ampliamente para tratar el asma desde que se describió por primera vez su eficacia en esta enfermedad. Actualmente sabemos que su uso está asociado con la aparición de efectos adversos (EA), como la osteoporosis o la insuficiencia suprarrenal. Este es un estudio observacional basado en la metodología Delphi. El cuestionario constaba de 4 bloques principales: generalidades de los GCS; tratamiento de mantenimiento; tratamiento en ciclos cortos, y EA.

**Materiales y métodos:**

Un panel de 48 expertos profesionales alergólogos y neumólogos respondieron a 2 rondas de un cuestionario de 68 enunciados.

**Resultados:**

Se consensuaron definiciones y hubo acuerdos sobre el uso apropiado de los GCS en el tratamiento del asma grave. Los expertos consensuaron que el uso de los GCS se debe minimizar en lo posible y estuvieron de acuerdo en un esquema de retirada gradual y progresivo en caso de terapias de mantenimiento. Además, destacaron la necesidad de estandarizar el procedimiento de control de la medida de GCS administrado tanto en ciclos cortos como en terapias de mantenimiento a fin de controlar mejor la carga acumulada de los pacientes que reciben dicho tratamiento y evitar la aparición de EA.

**Conclusiones:**

Este documento de consenso pretende aunar las recomendaciones de los expertos en el manejo del asma respaldadas por la evidencia científica con el objetivo de impulsar la reducción en el uso de GCS en el ámbito español.

## Graphical abstract

**Figure:**
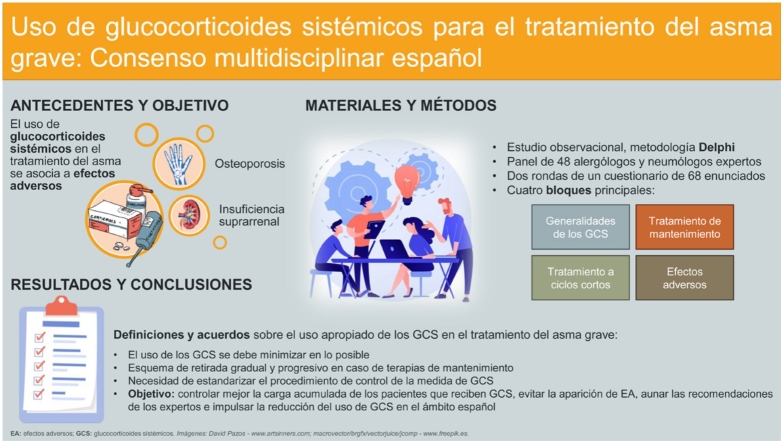

## Abstract (en)

**Background and aim:**

Since their effectiveness was initially demonstrated, oral corticosteroids (OCS) have been routinely used to treat asthma. We now know that their usage is linked to the development of side effects such osteoporosis and adrenal insufficiency. This is an observational study based on Delphi methodology. The questionnaire was divided into 4 sections: OCS generalities, maintenance treatment, short-term treatment, and adverse events.

**Materials and methods:**

Two rounds of a 68-item questionnaire were completed by a panel of 48 allergists and pneumologists.

**Results:**

Definitions were agreed upon, as was the proper use of OCS in the treatment of severe asthma. The experts agreed that the use of OCS should be minimized as much as possible and that in the event of maintenance treatments, a slow and progressive tapering strategy should be used. They also emphasized the importance of standardizing the technique for measuring the amount of SCG delivered in both cases.

**Conclusions:**

This consensus document attempts to bring together scientifically supported suggestions from specialists in the management of asthma to reduce the use of OCS in Spain.

## Introducción

El asma es una enfermedad inflamatoria crónica del tracto respiratorio cuyas características fisiopatológicas fundamentales son la hiperrespuesta bronquial y la obstrucción variable del flujo aéreo^1^. Aproximadamente 400 millones de personas en todo el mundo^2^ y casi 2 millones de adultos en España (> 5%) padecen asma^3^, ^4^, aunque se han descrito diferencias geográficas considerables en la prevalencia^5^.

Los objetivos del tratamiento del asma son lograr un buen control de los síntomas y minimizar el riesgo de muerte, el deterioro de la función pulmonar, las exacerbaciones y los efectos adversos (EA) del tratamiento relacionados con el asma^6^, ^7^. Con el objetivo de mejorar el control del asma y prevenir las exacerbaciones, las guías nacionales e internacionales recomiendan un tratamiento escalonado^7^, ^8^. A pesar de estas recomendaciones y de la disponibilidad de terapias efectivas, la proporción de pacientes con asma no controlada sigue siendo alta, debido principalmente a la falta de adhesión a la terapia de mantenimiento, una técnica de uso del inhalador deficiente, el uso excesivo de la terapia de alivio y a la presencia de comorbilidades^9^, ^10^.

Los glucocorticoides (GC) son inhibidores potentes de la inflamación y se utilizan para tratar una amplia variedad de afecciones inflamatorias y autoinmunes, incluida el asma. Para ejercer su efecto, los GC deben difundir a través de la membrana plasmática y unirse a su receptor a nivel citoplasmático. Una vez activado el complejo, distintos mediadores facilitan su translocación al núcleo, donde se generan efectos directos e indirectos sobre el control de la inflamación, dando lugar a la disminución de la expresión de distintos mediadores involucrados en mantener el proceso inflamatorio. Al mismo tiempo, provoca la disminución del reclutamiento y la activación de las células inflamatorias, el aumento de los receptores β2, la disminución de la permeabilidad microvascular y la producción de moco^11^.

Los GC sistémicos (GCS) se han utilizado para tratar el asma desde la década de 1950, cuando se describió por primera vez su eficacia en esta enfermedad^12^. Sin embargo, su uso está asociado con la aparición de EA, como la osteoporosis o fracturas osteoporóticas, la neumonía, las enfermedades cardiovasculares y cerebrovasculares, las cataratas, la apnea del sueño, la depresión o ansiedad, la diabetes tipo 2, el aumento de peso y la insuficiencia renal o insuficiencia suprarrenal (IS)^13^, ^14^, ^15^, ^16^, ^17^. En la tabla 1 se especifican los GCS disponibles, su potencia y duración de acción comparada con la hidrocortisona (molécula similar al corticosteroide endógeno) y los de más frecuente utilización en el paciente con asma^18^.

**Tabla 1:** Propiedades, dosis equivalentes e indicaciones terapéuticas de los corticosteroides sistémicos, en relación con hidrocortisona

	Dosis equivalente (mg)	Efecto antiinflamatorio en relación con la hidrocortisona	Duración de la acción (h)
*Acción corta*			
Hidrocortisona	20	1	8-12
Acetato de cortisona	25	0,8	8-12
*Acción intermedia*			
Prednisona	5	4	12-36
Prednisolona	4	5	12-36
Metilprednisolona	4	5	12-36
Triamcinolona	4	5	12-36
*Acción larga*			
Dexametasona	0,75	30	36-72
Betametasona	0,6	30	36-72

Aproximadamente el 5-10% de la población total con asma tiene un diagnóstico de asma grave^19^, y más de la mitad de los cuales tienen, además, un control deficiente de la enfermedad^5^, ^20^. El asma grave se asocia con una mayor morbilidad relacionada con el asma, mayores costos de atención médica, exacerbaciones más frecuentes y un mayor uso de GCS en comparación con el asma leve o moderada^21^, ^22^, ^23^. No hay suficientes datos de uso de GCS en España en diferentes poblaciones. Un estudio reciente mostró que, de media, un 30,2% de los pacientes con asma grave utilizó GCS de forma mantenida en el último año^24^.

A pesar de que la terapia complementaria con GCS de dosis baja a largo plazo se limita al escalón 5 de la Guía de la Iniciativa Global para el Asma (GINA)^7^ y al escalón 6 de la Guía Española para el Manejo del Asma (GEMA), se siguen utilizando para el tratamiento a largo plazo del asma grave, con un uso global estimado en 20-60%^13^. Además, hay que tener en cuenta el uso de los GCS asociado a las exacerbaciones o incluso al uso de otras enfermedades muchas veces concomitantes con el asma como, entre otras, la rinosinusitis crónica con poliposis nasal.

En los últimos años, las nuevas terapias biológicas han demostrado su eficacia tanto para reducir el uso de GCS como las tasas de exacerbación en pacientes con asma grave^25^, ^26^. En consecuencia, de acuerdo con las guías recientes, estas nuevas terapias se recomiendan como una alternativa para minimizar el riesgo de EA relacionados con los GCS en pacientes con asma grave^7^, ^8^.

Una revisión sistemática publicada por Cochrane (18 ensayos clínicos; *n* = 2.438 pacientes), concluyó que la evidencia sobre el mejor esquema de tratamiento con GCS es aún débil^27^. La ausencia de datos clínicos para desarrollar guías basadas en evidencia, junto con un algoritmo de tratamiento poco claro, un uso excesivo de GCS en la práctica clínica y la diversidad de entornos de atención médica que manejan pacientes con asma, ha motivado este estudio de consenso Delphi que tuvo como objetivo reunir a un grupo de expertos con conocimientos relevantes y experiencia clínica en el manejo de estos pacientes para generar una declaración de consenso sobre el uso más adecuado de los GCS en el asma.

## Materiales y métodos

### Diseño del estudio

*Este es un estudio observacional basado en la metodología Delphi. El panel de expertos que participaron en el estudio respondiendo las 2 rondas del cuestionario Delphi estaba formado por profesionales médicos alergólogos y neumólogos con amplia experiencia en el manejo de asma grave. El cuestionario fue desarrollado por el comité coordinador y los participantes respondieron a las 2 rondas del cuestionario tipo Delphi que fue suministrado de forma anónima mediante una plataforma interactiva.*

### Participantes y expertos del comité coordinador

Los expertos que fueron invitados a conformar el panel eran miembros de la Sociedad Española de Neumología y Cirugía Torácica (SEPAR) y de la Sociedad Española de Alergología e Inmunología Clínica (SEAIC). Los criterios de inclusión para la participación de los expertos a conformar el panel fueron: 1) que formasen parte de una de las 2 sociedades científicas mencionadas; 2) que tuvieran experiencia relevante en el manejo de pacientes con asma grave, con un mínimo de 5 años ejerciendo su especialidad, y 3) que el panel resultante tuviera la misma contribución de participantes de ambas sociedades científicas.

El comité coordinador fue conformado por 5 especialistas en los campos de neumología, alergología y endocrinología (2 integrantes de cada una de las sociedades SEPAR y SEAIC, y una integrante de la Sociedad Española de Endocrinología y Nutrición). Los expertos del comité fueron responsables del desarrollo del cuestionario Delphi, la revisión de los resultados de ambas rondas y la interpretación de sus resultados.

### Cuestionario

El comité coordinador, basándose en su experiencia, propuso una serie de enunciados preliminares para el desarrollo del cuestionario que, en una reunión virtual, fueron discutidos y revisados hasta conformar el cuestionario definitivo con 68 enunciados que se dividió en 4 bloques principales: 1) generalidades de los GCS; 2) tratamiento de mantenimiento; 3) ciclos cortos, y 4) EA. Todos los enunciados fueron revisados y validados por todos los integrantes del comité coordinador y, particularmente los referidos a desescalada y carga acumulada de los GCS fueron especialmente revisados por la especialista en el campo de la endocrinología.

### Consenso y análisis estadístico

El consenso se basó en el método de adecuación de la corporación RAND Healthcare y la Universidad de California en Los Ángeles^28^. En ambas rondas, se pidió a los participantes que calificaran cada enunciado en una escala Likert de 9 puntos para evaluar su acuerdo o desacuerdo (1 = totalmente en desacuerdo; 9 = totalmente de acuerdo)^29^. En la segunda ronda, los participantes revaluaron exclusivamente los enunciados para los que no se llegó a un consenso en la primera ronda.

Los enunciados se clasificaron como inapropiados, inciertos o apropiados cuando se registró una puntuación media de 1-3, 4-6 o 7-9, respectivamente. Se utilizó la desviación absoluta media de la mediana para medir la dispersión estadística. Se logró el consenso si al menos 2 tercios del panel puntuaron dentro del rango que contiene la mediana; de lo contrario, se consideró como ausencia de consenso. Sin embargo, se consideró controversia cuando más de un tercio de las puntuaciones individuales se encontraban dentro del rango opuesto al que contenía la mediana. Los datos se analizaron utilizando el paquete estadístico R versión 3.2.5 (R Core Team, 2020).

## Resultados

### Participantes y desarrollo del cuestionario

De los 50 expertos que fueron invitados a formar parte del panel, todos accedieron a participar y 48 (96,0%, 24 participantes de cada sociedad) completaron ambas rondas del cuestionario Delphi (anexo, tabla S 1). Las características demográficas del panel se describen en la tabla 2.

**Tabla 2:** Datos sociodemográficos de los expertos que conformaron el panel

*Comunidad Autónoma, n (%)*	
Andalucía	9 (19%)
Aragón	2 (4%)
Castilla-La Mancha	3 (6%)
Castilla y León	1 (2%)
Cataluña	6 (13%)
Comunidad de Madrid	14 (29%)
Comunidad Valenciana	4 (8%)
Extremadura	1 (2%)
Galicia	3 (6%)
Navarra	2 (4%)
País Vasco	2 (4%)
Región de Murcia	1 (2%)
*Años de especialidad, mediana (mín.; máx.)*	25 (10; 45)

El cuestionario fue acordado por los coordinadores durante un periodo de 2 meses (febrero a marzo del 2021).

### Resultados del Consenso (tabla 3)

#### Bloque 1: los glucocorticoides sistémicos en asma

Los panelistas mostraron su acuerdo acerca de todos los enunciados relacionados con el tipo de GCS más utilizados en el tratamiento del asma: prednisona y metilprednisolona. Además, hubo consenso sobre la falta de evidencia para la indicación de esteroides alternativos a la prednisona y sobre la hidrocortisona, siendo la última el tratamiento de elección como tratamiento sustitutivo en caso de IS. También se alcanzó un consenso sobre la necesidad de la unificación de la medida de la carga acumulativa de corticoides en equivalente de prednisona en la práctica clínica habitual.

**Tabla 3:** Resultados del cuestionario Delphi

		Apropiado	Acuerdo	Mediana	MAD-M
Bloque 1	Los glucocorticoides en asma				
P4	Los glucocorticoides que se usan en el tratamiento del asma son principalmente los de vida media intermedia (prednisona y metilprednisolona)	Apropiado	Acuerdo	9	0,62
P5	No hay evidencia suficiente para indicar que otros esteroides alternativos, como la dexametasona, ofrezcan alguna ventaja adicional sobre la prednisona	Apropiado	Acuerdo	8	0,94
P6	La hidrocortisona es una molécula similar al corticoide endógeno y el tratamiento de elección como tratamiento sustitutivo de la insuficiencia suprarrenal	Apropiado	Acuerdo	9	0,88
P7	Se debe unificar la carga acumulativa de corticoides en equivalente de prednisona	Apropiado	Acuerdo	8	0,69
P8	El uso de GCS puede influir sobre los biomarcadores del asma, por lo que se deben tener en cuenta al considerar el inicio de un tratamiento biológico.	Apropiado	Acuerdo	9	0,44
P9	El uso de GCS reduce el recuento de eosinófilos de forma dependiente de dosis hasta 12 semanas tras su suspensión.	Apropiado	Acuerdo	8	0,69

Bloque 2	Tratamiento de mantenimiento del asma con GCS				
*Definiciones*					
P11	Se considera tratamiento crónico el uso de corticoide sistémico durante 6 meses al año, independientemente de la dosis	Apropiado	Acuerdo	8	1,19
P12	Teniendo en cuenta su respuesta anterior, exprese su acuerdo con la siguiente afirmación: «Se considera tratamiento de mantenimiento con corticoides sistémicos una dosis fija a lo largo de 3 meses»	Apropiado	Acuerdo	8	0,81
P13	Se considera tratamiento crónico una dosis al año acumulada de ≥ 1 g de equivalente de prednisona, independientemente del tiempo de tratamiento	Apropiado	Acuerdo	7	1,5
P14	En el tratamiento de mantenimiento, se consideran dosis bajas de GCS, dosis ≤5 mg/día de equivalente de prednisona	Apropiado	Acuerdo	8	1,04
P15	Teniendo en cuenta que en las recomendaciones de la GINA se considera que dosis ≤7,5 mg/día de equivalente de prednisona constituyen dosis bajas de GCS en tratamientos de mantenimiento, exprese de nuevo su acuerdo para la siguiente afirmación: «En el tratamiento de mantenimiento, se consideran dosis bajas de GCS, dosis ≤ 7,5 mg/día de equivalente de prednisona»	Apropiado	Neutral	7	1,31
*Indicaciones*					
P16	Los GCS deben administrarse de forma mantenida como última opción terapéutica, cuando las demás acciones para el control del asma han fracasado	Apropiado	Acuerdo	9	0,69
P17	Se debe utilizar la mínima dosis eficaz posible de GCS y durante el mínimo tiempo posible	Apropiado	Acuerdo	9	0,23
P18	El uso de GCS a largo plazo no es apropiado en situaciones donde estén disponibles otras opciones de tratamiento	Apropiado	Acuerdo	9	0,19
P19	Si un paciente con asma controlada recibe una dosis acumulada de 0,5 g de equivalente de prednisona en un año, es recomendable plantearse alternativas terapéuticas	Apropiado	Acuerdo	8	0,98
P20	Si un paciente con asma controlada recibe una dosis acumulada de 1 g de equivalente de prednisona en un año, es recomendable plantearse alternativas terapéuticas	Apropiado	Acuerdo	9	0,38
P21	Se debe promover el uso tratamientos biológicos en aquellos pacientes con asma grave no controlada que sufran ≥2 exacerbaciones que precisen GCS o ingreso hospitalario, a pesar de haber optimizado el tratamiento	Apropiado	Acuerdo	9	0,62
P22	Se debe promover el uso de tratamientos biológicos en cualquier paciente con asma grave no controlada en tratamiento con GCS de mantenimiento	Apropiado	Acuerdo	9	0,62
P23	Con el objetivo de suspender o reducir al mínimo la dosis de GCS, se debe considerar el uso de fármacos biológicos en el caso de asma con inflamación tipo T2	Apropiado	Acuerdo	9	0,21
P24	Se debe evitar una dosis > 40 mg/día de equivalente de prednisona debido al alto riesgo de efectos secundarios añadidos	Apropiado	Acuerdo	9	0,31
P25	Se debe evitar una dosis >30 mg/día de equivalente de prednisona debido al alto riesgo de efectos secundarios añadidos	Apropiado	Acuerdo	9	0,58
P26	Se debe evitar una dosis > 20 mg/día de equivalente de prednisona debido al alto riesgo de efectos secundarios añadidos	Apropiado	Acuerdo	9	1,1
P27	Se debe evitar una dosis > 10 mg/día de equivalente de prednisona debido al alto riesgo de efectos secundarios añadidos	Apropiado	Acuerdo	8	1,56
*Seguimientoy duración*					
P28	Desde el primer año de tratamiento de mantenimiento con GCS, los pacientes deben monitorizarse cada 3-6 meses la presión arterial, el sobrepeso y la glucemia	Apropiado	Acuerdo	9	0,54
P29	Desde el primer año de tratamiento de mantenimiento con GCS, los pacientes deben monitorizarse de forma anual la posible afectación del sistema óseo, ocular y digestivo	Apropiado	Acuerdo	9	0,73
P30	La efectividad de los GCS debe evaluarse periódicamente considerando el objetivo terapéutico que motivó su indicación	Apropiado	Acuerdo	9	0,29
P31	Si el objetivo de la indicación de GCS es reducir el número de exacerbaciones, la respuesta debe evaluarse en un periodo no inferior a 6 meses de iniciado el tratamiento	Apropiado	Acuerdo	8	1,15
P32	Si el objetivo de la indicación de GCS es la mejoría de síntomas, la respuesta debe evaluarse entre 1-3 meses de iniciado el tratamiento	Apropiado	Acuerdo	8	0,75
P33	Debe considerarse la retirada de GCS cuando se constata que el tratamiento ha sido efectivo, y el asma se ha controlado	Apropiado	Acuerdo	9	0,67
P34	Debe considerarse la retirada de GCS cuando se constata que el tratamiento no es efectivo	Apropiado	Acuerdo	9	0,48
*Desescalada*					
P35	En los pacientes que durante un tiempo prolongado se han tratado con dosis fijas altas de GCS, se debe ser cauto en la retirada con el fin de evitar la pérdida del control del asma o la aparición de una insuficiencia suprarrenal	Apropiado	Acuerdo	9	0,15
P36	En pacientes en desescalada de un tratamiento con GCS, una determinación de cortisol plasmático basal a las 8:00 < 5 μg/dl o 100 mmol/l indica una insuficiencia suprarrenal	Apropiado	Acuerdo	9	0,77
P37	La duración del tratamiento con GCS y la presencia de efectos adversos son factores importantes a tener en cuenta en la estrategia de desescalada	Apropiado	Acuerdo	9	0,27
P38	El riesgo de insuficiencia suprarrenal es mayor con dosis >5 mg/día de equivalente de prednisona durante > 4 semanas	Apropiado	Acuerdo	8	0,71
P39	Para reducir la dosis de GCS por debajo de 5 mg/día de equivalente de prednisona, los pacientes que hayan recibido una dosis acumulada de GCS superior a 2 g de equivalente de prednisona en el último año deben realizarse un estudio de cortisol plasmático a las 8:00	Apropiado	Acuerdo	9	0,67
P40	Cuando la dosis diaria de GCS de un paciente en tratamiento de mantenimiento supere los 20 mg/día se debe reducir la dosis en 5 mg/día semanalmente hasta llegar a la dosis diaria de 20 mg	Apropiado	Acuerdo	8	1,25
P41	Cuando la dosis diaria de GCS de un paciente en tratamiento de mantenimiento supere los 10 mg/día hasta los 20 mg/día se debe reducir la dosis en 5 mg/día quincenalmente hasta llegar a la dosis diaria de 10 mg	Apropiado	Acuerdo	8	1,17
P42	Cuando la dosis diaria de GCS de un paciente en tratamiento de mantenimiento supere los 7,5 mg/día hasta los 10 mg/día se debe reducir la dosis en 2,5 mg/día quincenalmente hasta llegar a la dosis diaria de 7,5 mg	Apropiado	Acuerdo	8	1,23
P43	Cuando la dosis diaria de GCS de un paciente en tratamiento de mantenimiento supere los 5 mg/día hasta los 7,5 mg/día se debe reducir la dosis en 2,5 mg/día mensualmente hasta llegar a la dosis diaria de 5 mg	Apropiado	Acuerdo	8	1,35

Bloque 3	Los GCS en ciclos cortos para el tratamiento de la crisis de asma				
*Definiciones*					
P45	Teniendo en cuenta que el panel consensuó como apropiado en la primera ronda el siguiente enunciado «Un ciclo corto de GCS consiste en una dosis diaria de 0,5-1 mg/kg del peso ideal por día de equivalente de prednisona, con un máximo de 40-50 mg, durante 5-7 días», exprese de nuevo su acuerdo para la siguiente afirmación: un ciclo corto de GCS consiste en una dosis diaria fija de 50 mg de equivalente de prednisona en forma de dosis única matutina, durante 5-7 días	Apropiado	Acuerdo	8	0,71
P46	Un ciclo corto de GCS consiste en una dosis diaria de 0,5-1 mg/kg del peso ideal por día de equivalente de prednisona, con un máximo de 40-50 mg, durante 5-7 días	Apropiado	Acuerdo	8	0,92
P47	La duración estándar de un ciclo corto de GCS eficaz es de 5 a 7 días	Apropiado	Acuerdo	8	1,19
P48	Teniendo en cuenta que el panel consensuó como apropiados en la primera ronda los enunciados «La duración estándar de un ciclo corto de GCS eficaz es de 5 a 7 días» y «Un ciclo corto de GCS no debe superar los 15 días, con una duración ideal de 7 días» exprese de nuevo su nivel de acuerdo con la siguiente afirmación: «Un ciclo corto de GCS es cualquiera que resulte inferior a 30 días»	Inapropiado	Desacuerdo	2	1,4
P49	Un ciclo corto de GCS no debe superar los 15 días, con una duración ideal de 7 días	Apropiado	Acuerdo	8	1,12
*Indicaciones*					
P50	Un ciclo corto de GCS constituye el tratamiento específico de las exacerbaciones graves	Apropiado	Acuerdo	8	0,83
P51	En caso de una crisis moderada a grave, el uso de un ciclo de GCS acelera la resolución de las crisis y previene las recaídas	Apropiado	Acuerdo	8	0,79
P52	El tratamiento hospitalario de las exacerbaciones graves del asma con GCS debe iniciarse dentro de la primera hora de la presentación de la exacerbación	Apropiado	Acuerdo	8	0,94
P53	Un ciclo corto de GCS (0,5-1 mg/kg del peso ideal por día de prednisona o equivalente hasta un máximo de 50 mg) es eficaz para reducir las exacerbaciones, la necesidad de atención hospitalaria por exacerbación y las dosis de β-agonistas de rescate	Apropiado	Acuerdo	8	0,98
P54	Si su administración es posible, la terapia oral de GCS es preferible a la administración intramuscular o intravenosa	Apropiado	Acuerdo	8	0,73
P55	No se puede establecer una dosis fija ideal de GCS para ciclo corto	Apropiado	Acuerdo	8	1,42
P56	Añadir GCS al tratamiento con broncodilatadores mejora la evolución de las exacerbaciones en pacientes con asma no eosinofílica	Apropiado	Acuerdo	7	1,33
P57	En el inicio del tratamiento de mantenimiento en pacientes con asma moderada a grave, añadir un ciclo corto de GCS no proporciona ningún beneficio clínico significativo. (siendo 1 «totalmente en desacuerdo» y 9 «totalmente de acuerdo»)	Incierto	Desacuerdo	6	1,54
P58	Los GCS no deben utilizarse como medicación de rescate	Apropiado	Acuerdo	8	1,21
P59	Los GCS no se deben autoadministrar por parte del paciente sin indicación médica	Apropiado	Acuerdo	9	0,56
P60	El tipo de exacerbación (eosinofílica o no eosinofílica) puede influir en respuesta al tratamiento con GCS	Apropiado	Acuerdo	8	1,04
*Seguimiento y duración*					
P61	Los pacientes adultos con un plan personal de manejo del asma y tratamiento con GCI pueden reducir la duración del ciclo a 5 días	Apropiado	Acuerdo	8	0,69
P62	Un ciclo corto de GCS no precisa retirada gradual si el tratamiento es inferior a 2 semanas	Apropiado	Acuerdo	8	1,17
P63	Se recomienda evaluar el desarrollo de insuficiencia suprarrenal en aquellos pacientes que reciban > 4 ciclos cortos de GCS por año	Apropiado	Acuerdo	8	1,15

Bloque 4	Efectos adversos y carga acumulada de GCS				
P65	La exposición continua a GCS se asocia significativamente con un mayor riesgo de efectos adversos como infecciones, afecciones cardiovasculares, alteraciones metabólicas, afecciones psiquiátricas, afecciones oculares, afecciones gastrointestinales y complicaciones óseas	Apropiado	Acuerdo	9	0,15
P66	Los efectos adversos relacionados con el uso de la terapia de mantenimiento con GCS dependen de la dosis	Apropiado	Acuerdo	9	0,56
P67	Los efectos adversos relacionados con el uso de la terapia de mantenimiento con GCS dependen de la dosis y son acumulativos en el tiempo	Apropiado	Acuerdo	9	0,54
P68	Se debe consignar en la historia clínica del paciente la carga acumulada de GCS anual	Apropiado	Acuerdo	9	0,56
P69	Ciclos cortos de GCS pueden asociarse con efectos adversos y cada prescripción de GCS supone una carga acumulativa, independientemente de la dosis y duración	Apropiado	Acuerdo	8	0,92
P70	Cualquier paciente que supere un uso acumulado de GCS > 1 g/año de equivalente de prednisona, debería ser correctamente revaluado para identificar posibles áreas de mejora en el manejo del asma	Apropiado	Acuerdo	9	0,4
P71	Dosis acumuladas de 1,0 g de prednisona o equivalente en un año aumentan significativamente el riesgo de efectos adversos	Apropiado	Acuerdo	9	0,54
P72	Dosis acumuladas de 2,5 g de prednisona o equivalente en un año aumentan significativamente el riesgo de efectos adversos	Apropiado	Acuerdo	9	0,42
P73	Se considera un riesgo de padecer efectos adversos con > 4 ciclos o > 30 días al año de tratamiento con GCS	Apropiado	Acuerdo	9	0,71
P74	El tratamiento con GCS se asocia con un aumento del gasto sanitario, en parte debido a la necesidad de resolver los efectos adversos relacionados	Apropiado	Acuerdo	8	0,88

MAD-M: desviación absoluta desde la mediana (por sus siglas en inglés).

##### Enmascaramiento de biomarcadores

El consenso entre los panelistas en relación con el impacto del tratamiento con GCS en los biomarcadores del asma se observó en todos los enunciados planteados. Hubo acuerdo entre los panelistas acerca de la influencia del tratamiento con GCS sobre los biomarcadores actuales del asma, como la reducción del recuento de eosinófilos, dependiente de dosis y hasta 12 semanas después de la suspensión del tratamiento, por lo que es recomendable considerar esta posibilidad y valorar los criterios de administración de tratamientos biológicos.

#### Bloque 2: tratamiento crónico o de mantenimiento del asma con GCS

##### Definiciones

Acerca del tratamiento crónico o de mantenimiento del asma con GCS, hubo consenso entre los panelistas en la mayoría de los ítems planteados (5/6 ítems) y solo en uno de ellos el resultado fue neutral. El panel de expertos consideró como tratamiento crónico con GCS las 2 situaciones clínicas siguientes: los tratamientos de GCS con duración de 6 meses al año, independientemente de la dosis; y, por otra parte, los tratamientos con dosis acumuladas ≥1 g de equivalente de prednisona al año, independientemente de la duración del tratamiento. Por otra parte, no se obtuvo consenso entre los panelistas en cuanto a que dosis ≤ 7,5 mg/día de equivalente de prednisona para el tratamiento de mantenimiento se deban considerar dosis bajas de GCS. Sin embargo, los panelistas acordaron que en los tratamientos de mantenimiento con GCS, dosis ≤ 5 mg/día de equivalente de prednisona pueden considerarse bajas.

##### Indicación

Los panelistas estuvieron de acuerdo en todos los enunciados relacionados con la indicación del tratamiento de mantenimiento de GCS. Hubo acuerdo en que los GCS deben administrarse de forma mantenida como última opción terapéutica, después de probar el resto de las opciones para el control del asma y que estas hayan fracasado. Además, se debería utilizar la mínima dosis eficaz y durante el mínimo tiempo posible. Estuvieron de acuerdo también en que el uso de GCS a largo plazo no es apropiado en situaciones donde estén disponibles otras opciones de tratamiento. Por otra parte, hubo acuerdo en cuanto a que para un paciente con asma controlada que recibe una dosis acumulada de ≥ 0,5 g de equivalente de prednisona en un año, es recomendable plantear alternativas terapéuticas.

También se observó acuerdo en que se debe promover el uso de tratamientos biológicos en aquellos pacientes con asma grave no controlada que presentan 2 o más exacerbaciones que precisen GCS o ingreso hospitalario, a pesar de haber optimizado el tratamiento y según su fenotipificación. Igualmente se debe promover el uso de tratamientos biológicos en cualquier paciente con asma grave no controlada en tratamiento con GCS de mantenimiento. Además, se debe considerar el uso de fármacos biológicos en el caso de asma con inflamación tipo T2, con el objetivo de suspender o reducir al mínimo la dosis de GCS. Finalmente, en esta sección hubo acuerdo en cuanto a que se debe evitar cualquier dosis mayor a > 10 mg/día de equivalente de prednisona debido al alto riesgo de efectos secundarios añadidos.

##### Seguimiento y duración

En cuanto al seguimiento y la duración de los tratamientos de mantenimiento con GCS, los panelistas mostraron su consenso en todos los ítems planteados. Hubo consenso en cuanto a que, desde el primer año de tratamiento de mantenimiento con GCS, se debe monitorizar cada 3-6 meses la presión arterial, el sobrepeso y la glucemia de los pacientes, y, de forma anual, se debe comprobar la posible afectación del sistema óseo, ocular y digestivo. Además, hubo acuerdo en que la efectividad de los GCS debe evaluarse periódicamente considerando el objetivo terapéutico que motivó su indicación. En caso de que el objetivo de la indicación de GCS fuera reducir el número de exacerbaciones, la respuesta debe evaluarse en un periodo no inferior a 6 meses de iniciado el tratamiento; y si el objetivo de la indicación de GCS es la mejoría de los síntomas, la respuesta debe evaluarse entre 1-3 meses de iniciado el tratamiento. También los panelistas estuvieron de acuerdo en que se debe considerar la retirada de GCS cuando se constata que el tratamiento ha sido efectivo, y el asma se ha controlado, pero también cuando se constata que el tratamiento no es efectivo.

##### Procedimiento de retirada de los GCS: desescalada

En cuanto a la desescalada, hubo consenso entre los panelistas en todos los ítems planteados. Hubo acuerdo en que en los pacientes que han recibido dosis fijas altas de GCS durante un tiempo prolongado, se debe ser cauto en la retirada del tratamiento con el fin de evitar la pérdida del control del asma o la aparición de una IS. Estuvieron de acuerdo en cuanto a que para reducir la dosis de GCS por debajo de 5 mg/día de equivalente de prednisona, los pacientes que hayan recibido una dosis acumulada de GCS superior a 2 g de equivalente de prednisona en el último año deben realizarse un estudio de cortisol plasmático a las 8:00 de la mañana, y que una determinación de cortisol plasmático basal a las 8:00 de la mañana menor a 5 μg/dl o 100 mmol/l indica una IS para un paciente en desescalada de un tratamiento de GCS mantenido.

Los panelistas mostraron su acuerdo respecto a que la duración del tratamiento con GCS y la presencia de EA son factores importantes para tener en cuenta en la estrategia de desescalada, y que el riesgo de IS es mayor con dosis mayores de 5 mg/día de equivalente de prednisona durante más de 4 semanas.

En cuanto a la estrategia de reducción de la dosis diaria de GCS, los panelistas estuvieron de acuerdo en que:

– Cuando la dosis diaria de GCS de un paciente en tratamiento de mantenimiento supere los 20 mg/día, se debe reducir la dosis en 5 mg/día de forma semanal hasta llegar a la dosis diaria de 20 mg.
– Cuando la dosis diaria de GCS de un paciente en tratamiento de mantenimiento supere los 10 mg/día hasta los 20 mg/día, se debe reducir la dosis en 5 mg/día de forma quincenal hasta llegar a la dosis diaria de 10 mg.
– Cuando la dosis diaria de GCS de un paciente en tratamiento de mantenimiento supere los 7,5 mg/día hasta los 10 mg/día, se debe reducir la dosis en 2,5 mg/día de forma quincenal hasta llegar a la dosis diaria de 7,5 mg.
– Cuando la dosis diaria de GCS de un paciente en tratamiento de mantenimiento supere los 5 mg/día hasta los 7,5 mg/día, se debe reducir la dosis en 2,5 mg/día de forma mensual hasta llegar a la dosis diaria de 5 mg.

#### Bloque 3: los GCS en ciclos cortos para el tratamiento de la crisis de asma

##### Definiciones para los ciclos cortos de GCS

Respecto a las definiciones planteadas sobre los ciclos cortos para el tratamiento de la crisis de asma, se alcanzó el consenso sobre los 5 planteamientos propuestos, en 4 los panelistas estuvieron de acuerdo y en uno, en desacuerdo. Hubo acuerdo entre los panelistas sobre la definición de un ciclo corto de GCS, que consiste en una dosis diaria fija de 50 mg de equivalente de prednisona en forma de dosis única matutina, durante 5-7 días, o bien, una dosis diaria de 0,5-1 mg/kg del peso ideal por día de equivalente de prednisona, con un máximo de 40-50 mg, durante 5-7 días. Además, la duración estándar de un ciclo corto de GCS eficaz se consideró entre los 5 a 7 días y no debe superar los 15 días. Por otra parte, hubo consenso en el desacuerdo sobre que un ciclo corto de GCS es cualquiera que resulte inferior a 30 días.

##### Indicaciones de los ciclos cortos de GCS

En cuanto a las indicaciones de los ciclos cortos de GCS, hubo consenso entre los panelistas en la mayoría de los ítems planteados (10/11 ítems) y este consenso no se observó en uno de los ítems en que se evidenció un desacuerdo incierto.

Hubo acuerdo en cuanto a que un ciclo corto de GCS constituye el tratamiento específico de las exacerbaciones graves y, en caso de una crisis moderada a grave, el uso de un ciclo de GCS acelera la resolución de las crisis y previene las recaídas. Además, los panelistas consensuaron su acuerdo en cuanto a que el tratamiento hospitalario de las exacerbaciones graves del asma con GCS debe iniciarse dentro de la primera hora de la presentación de la exacerbación y que un ciclo corto de GCS (0,5-1 mg/kg del peso ideal por día de prednisona o equivalente hasta un máximo de 50 mg) es eficaz para reducir las exacerbaciones, la necesidad de atención hospitalaria por exacerbación y las dosis de β-agonistas de rescate. En cuanto a la administración, los panelistas estuvieron de acuerdo en que, si es posible, la terapia oral de GCS es preferible a la administración intramuscular o intravenosa, en que no se puede establecer una dosis fija ideal de GCS para ciclo corto, en que los GCS no deben considerarse una medicación de rescate, y en que los GCS no se deben autoadministrar por parte del paciente sin indicación médica. Además, consensuaron su acuerdo en que añadir GCS al tratamiento con broncodilatadores mejora la evolución de las exacerbaciones en pacientes con asma no eosinofílica, y en que el tipo de exacerbación (eosinofílica o no eosinofílica) puede influir en la respuesta al tratamiento con GCS.

Por otra parte, los panelistas expresaron su desacuerdo incierto en cuanto a que, en el inicio del tratamiento de mantenimiento en pacientes con asma moderada a grave, añadir un ciclo corto de GCS no proporciona ningún beneficio clínico significativo.

##### Seguimiento y duración de los ciclos cortos de GCS

En cuanto al seguimiento y duración de los ciclos cortos de GCS, los panelistas consensuaron su acuerdo para todos los ítems planteados. Hubo acuerdo en cuanto a que los pacientes adultos con un plan personal de manejo del asma y en tratamiento con GCS pueden reducir la duración del ciclo a 5 días, en que un ciclo corto de GCS no precisa retirada gradual si el tratamiento es inferior a 2 semanas, y en que se recomienda evaluar el desarrollo de IS en aquellos pacientes que reciban más de 4 ciclos cortos de GCS por año.

#### Bloque 4: efectos adversos y carga acumulada de GCS

Sobre los EA y la carga acumulada de los tratamientos de GCS, hubo consenso entre los panelistas sobre todos los ítems planteados. Los panelistas estuvieron de acuerdo en que la exposición continua a GCS se asocia significativamente con un mayor riesgo de EA como infecciones, afecciones cardiovasculares, psiquiátricas, oculares y gastrointestinales, alteraciones metabólicas y complicaciones óseas. También estuvieron de acuerdo en que los EA relacionados con el uso de la terapia de mantenimiento con GCS dependen de la dosis y son acumulativos en el tiempo. Hubo acuerdo sobre la importancia de consignar en la historia clínica del paciente la carga acumulada de GCS anual. También estuvieron de acuerdo en que los ciclos cortos de GCS pueden asociarse con EA y cada prescripción de GCS supone una carga acumulativa, independientemente de la dosis y duración y en que cualquier paciente que supere un uso acumulado de GCS mayor a 1 g/año de equivalente de prednisona, debería ser correctamente revaluado para identificar posibles áreas de mejora en el manejo del asma.

Acerca de la carga acumulada, los expertos estuvieron de acuerdo en que dosis acumuladas ≥ 1,0 g de prednisona o equivalente en un año aumentan el riesgo de EA de forma significativa. Además, mostraron su acuerdo en cuanto a que más de 4 ciclos o más de 30 días al año de tratamiento con GCS se deben considera un riesgo de padecer EA. También estuvieron de acuerdo en que el tratamiento con GCS se asocia con un aumento del gasto sanitario, en parte debido a la necesidad de resolver los EA relacionados.

## Discusión

### Los glucocorticoides sistémicos en asma

Los resultados de varios estudios de investigación clínica indican una posible resolución rápida de los síntomas y una reducción de las tasas de su recurrencia entre los pacientes tratados con GCS. Además, varias revisiones sistemáticas han demostrado una reducción de las recaídas sintomáticas, la reducción del uso de agonistas β2 adrenérgicos de acción corta (SABA, por sus siglas en inglés) y la reducción de hospitalizaciones entre los pacientes tratados con GCS^27^, ^30^. Adicionalmente, las guías de consenso aún recomiendan el tratamiento de mantenimiento con dosis bajas de GCS durante largos periodos en asma grave no controlada con otras alternativas terapéuticas^7^, ^31^. Esto ha contribuido a considerar los GCS como el tratamiento preferido en las exacerbaciones del asma en los últimos 60 años^12^.

Actualmente existe una gran variabilidad en la dosis y duración de tratamientos con GCS utilizados para las crisis asmáticas y no se conoce qué régimen tiene mayores probabilidades de mejorar los síntomas y minimizar los EA^27^.

La prednisona tiene un coste relativamente bajo y es el GCS de mayor uso internacional. El panel de expertos consultado mostró su acuerdo en cuanto a que tanto la prednisona como la metilprednisolona son los GCS más utilizados en el tratamiento del asma en la práctica clínica en España y en que, actualmente, no hay pruebas suficientes para indicar que otros esteroides alternativos, como la dexametasona, ofrezcan ninguna ventaja sobre la prednisona^27^.

### Enmascaramiento de biomarcadores

Es conocido que la terapia con GCS reduce el recuento de diversos biomarcadores utilizados como referencia en el diagnóstico y manejo del paciente asmático, especialmente el recuento de eosinófilos en sangre periférica y esputo, aunque son escasos los trabajos que evalúen la magnitud de esta reducción y la cinética de recuperación tras la suspensión del tratamiento en el contexto de la práctica clínica real. En un trabajo publicado en 2019 por Ortega et al*.*^32^ se cuantificó el descenso de eosinófilos en sangre periférica entre 30% y 36% tras un mes de tratamiento con GCS como terapia de mantenimiento (media de 35 mg/día de equivalente de prednisona), dependiendo de la eosinofilia inicial, siendo mayor el descenso con niveles de partida más altos. Tras 3 meses de tratamiento con GCS, la reducción de eosinofilia se mantuvo en cifras similares y después de 12 semanas de interrumpir su administración, los eosinófilos sanguíneos no habían alcanzado aún sus niveles basales^32^.

La administración de ciclos cortos de GCS para el tratamiento de exacerbaciones asmáticas también produce diferentes alteraciones de los biomarcadores. Semprini et al*.*^33^ evaluaron el cambio en los biomarcadores de inflamación T2 tras exacerbaciones graves y observaron que los eosinófilos en sangre presentaron los niveles más bajos en el primer día de tratamiento y llegaron a sus niveles de referencia la segunda semana tras su suspensión. Los niveles plasmáticos de periostina no descendieron hasta la semana, con recuperación a las 4 semanas. El fracción exhalada de óxido nítrico (FeNO, por sus siglas en inglés) alcanzó su nivel mínimo a las 2 semanas, con recuperación casi completa de los niveles de referencia a las 8 semanas. En contraste, los niveles de IgE sérica fueron mayores que sus niveles de referencia en las primeras 24 h tras la exacerbación y permanecieron elevados hasta la segunda semana. Finalmente, todos los biomarcadores alcanzaron un nivel estable en torno a las 4-8 semanas después del inicio del tratamiento de la exacerbación con GCS^33^.

Por lo tanto, antes de tomar decisiones sobre la indicación de un determinado fármaco biológico basado en los niveles de eosinofilia, es necesario valorar la administración previa de corticoides sistémicos hasta 12 semanas antes, lo que está bien instaurado en la práctica clínica habitual española, según las respuestas del panel de expertos consultado.

### Tratamiento crónico o de mantenimiento del asma con GCS

#### Definiciones sobre los tratamientos crónicos o de mantenimiento

En el momento actual no existe un acuerdo unánime sobre lo que se entiende por tratamiento crónico o de mantenimiento con GCS ya que, además de una dosis diaria, algunos autores consideran que una dosis acumulada anual también puede ser considerada como tratamiento crónico, aunque se reciba de forma intermitente^34^, ^35^, ^36^.

El panel de expertos consultado en este trabajo consideró que, en la práctica habitual, un tratamiento de mantenimiento es aquel que dura de 6 meses a un año, independientemente de la dosis, o bien, aquellos tratamientos con dosis acumuladas ≥ 1 g de equivalente de prednisona, independientemente de la duración del tratamiento. Sin embargo, hasta el momento se desconoce cuál es el esquema de tratamiento con GCS más adecuado tanto en las crisis como de mantenimiento^27^. En este sentido, los panelistas estimaron que en los tratamientos de mantenimiento con GCS, se pueden considerar como bajas dosis ≤ 5 mg/día de equivalente de prednisona, pero no así dosis ≤ 7,5 mg/día de equivalente de prednisona, a pesar de que las recomendaciones de la GINA así lo indican. Esto podría señalar que la concienciación sobre la necesidad de reducir los tratamientos de GCS al mínimo posible se está haciendo extensiva a la práctica clínica española. La prescripción de GCS se realiza sobre todo cuando la persistencia de síntomas y las exacerbaciones son los responsables de la falta de control del asma, teniendo un papel menos preponderante en esta prescripción la obstrucción del flujo aéreo, especialmente cuando esta es aislada^13^, ^37^. Sin embargo, no existen estudios que relacionen alteraciones en un determinado dominio en el control del asma con un mayor uso de GCS.

#### Indicación del tratamiento crónico o de mantenimiento

La administración de GCS de mantenimiento ha sido una práctica habitual en pacientes con asma grave no controlada, a pesar de la adecuada adhesión al tratamiento máximo optimizado y actuación sobre los agravantes y comorbilidades^7^, ^21^, ^22^, ^34^, ^38^, ^39^. No obstante, desde hace años muchos estudios alertan que las dosis acumuladas de GCS se asocian con una gran variedad de EA^13^, ^18^, ^22^, ^35^, ^40^, ^41^. Además, el uso a largo plazo también se asocia con un aumento del riesgo de mortalidad, una peor calidad de vida y un aumento del consumo de recursos y de costes sanitarios^22^, ^38^, ^42^, ^43^, ^44^.

Por todo ello, los panelistas consultados estuvieron de acuerdo en que los GCS deben administrarse de forma mantenida como última opción terapéutica, después de probar el resto de las opciones para el control del asma y que estas hayan fracasado. Además, consideraron que se debe utilizar la mínima dosis eficaz y durante el mínimo tiempo posible, coincidiendo con las recomendaciones de las guías más usadas en España^7^, ^8^.

En el presente trabajo también se llegó a un consenso en que el uso de GCS a largo plazo no es apropiado en situaciones donde estén disponibles otras opciones de tratamiento, siendo recomendable plantear alternativas terapéuticas para los pacientes con asma controlada que reciben una dosis acumulada de > 0,5 g/año de equivalente de prednisona, puesto que existe evidencia que soporta que deben administrarse a la dosis más baja eficaz y durante el mínimo tiempo posible^45^, ^46^, ^47^.

El uso precoz de ciclos de GCS en la agudización asmática está apoyado por todas las guías^7^, ^8^. Sin embargo, no se dispone de guías basadas en la evidencia para la indicación de los GCS de mantenimiento. Los panelistas consultados estuvieron de acuerdo en que se debe evitar cualquier dosis mayor a 10 mg/día de equivalente de prednisona debido al alto riesgo de efectos secundarios añadidos. *Otros* consensos de expertos internacionales consideraron, por una parte, que ≤ 5 mg/día sería una dosis aceptable, que equivaldría a una dosis acumulada de 1,8 g/año^36^, o bien no alcanzaron acuerdo sobre si la dosis mayor a 5 mg/día se considera dosis alta siendo necesaria más evidencia sobre este aspecto^48^.

Afortunadamente, los avances en el conocimiento de los mecanismos fisiopatológicos del asma han permitido disponer de fármacos biológicos para el asma con inflamación tipo T2 que permiten suspender o reducir considerablemente la dosis de GCS, afirmación que obtuvo el consenso de acuerdo con el panel de expertos consultado en este trabajo^30^, ^49^. Relacionado con esto, es un pensamiento extendido el que no lograr una reducción ≥ 50% en los GCS indica un fracaso de la terapia biológica y puede justificar un cambio de estrategia^36^.

La GINA restringe la indicación de GCS a largo plazo añadido al tratamiento habitual al escalón 5 y lo posiciona después del ensayo con otros tratamientos (p. ej., tiotropio y biológicos)^5^. Por su parte, la GEMA 5.0 considera los GCS como la última opción terapéutica en el asma, cuando hayan fracasado otras opciones como la triple terapia y los fármacos biológicos en los casos con enfermedad fenotipo T2^50^. Otros consensos de expertos internacionales mostraron su acuerdo con las recomendaciones de la GINA en este sentido. Con respecto al uso apropiado de GCS, de la misma forma que en el presente documento, otros paneles de expertos internacionales consideraron que el uso a largo plazo no es apropiado en situaciones donde estén disponibles otras opciones de tratamiento^36^. Aun así, sigue habiendo cierta heterogeneidad en la práctica clínica relacionada con los tratamientos de GCS en mantenimiento para el tratamiento del asma.

Dado que el asma es una enfermedad con muchos fenotipos, la respuesta a los corticoides es variable^51^, ^52^. Los pacientes respondedores a GCS suelen tener inflamación tipo T2, mientras que el asma no-T2 suele tener pobre respuesta a GCS, incluso a dosis altas. Es decir que, es probable que muchos pacientes que no responden a los GCS tampoco respondan a los tratamientos biológicos dirigidos contra células o vías fisiopatológicas implicadas en la inflamación T2^30^, ^49^, y por ello debería evaluarse la respuesta entre los 3 y 6 meses valorando síntomas, calidad de vida, exacerbaciones, función pulmonar y efectos secundarios, sustentando la decisión de mantener el tratamiento crónico con GCS cuando los beneficios superen los riesgos.

#### Seguimiento y duración en el tratamiento crónico o de mantenimiento

Teniendo en cuenta que el uso de GCS en mantenimiento en el tratamiento del asma debe minimizarse en lo posible, para establecer el intervalo de valoración de la respuesta, deben de tenerse en cuenta una serie de premisas. En primer lugar, la base del tratamiento con GCS en el asma es la inflamación eosinofílica asociada a esta enfermedad^47^. Además, la respuesta a los GCS varía considerablemente entre pacientes^53^ y existe evidencia que apunta a que entre el 25-35% de los pacientes pueden ser resistentes a este tratamiento^54^, ^55^. Finalmente, es importante considerar el objetivo terapéutico por el cual se ha indicado el tratamiento de mantenimiento con GCS y el alto índice de EA que se asocian al tratamiento con GCS^17^. En este sentido, el panel de expertos consultado consensuó su acuerdo en que, si la indicación es reducir el número de exacerbaciones, la respuesta deberá evaluarse en un periodo no inferior a 6 meses y, si la indicación ha sido la mejoría de síntomas, la respuesta puede evaluarse entre 1-3 meses una vez iniciado el tratamiento, coincidiendo con otros paneles de expertos internacionales^48^. Adicionalmente, los panelistas del presente trabajo estuvieron de acuerdo en que es importante considerar la retirada de GCS cuando se constata que el tratamiento ha sido efectivo, y el asma se ha controlado, pero también cuando se constata que el tratamiento no es efectivo.

Independientemente de la valoración de la respuesta terapéutica, los pacientes tratados con GCS deben visitarse periódicamente con base en la prevención de posibles EA, como sobrepeso, diabetes, dislipidemia, hipertensión, glaucoma, osteoporosis, cataratas, trastornos digestivos o trastornos neuropsiquiátricos. Así, los expertos consultados mostraron su acuerdo en cuanto a que, al menos el primer año de su administración y probablemente de forma evolutiva, cada 3-6 meses debería monitorizarse la presión arterial, el sobrepeso, y la glucemia^18^. En relación con las posibles comorbilidades asociadas, el panel también consideró que debería monitorizarse, al menos de forma anual, la posible afectación del sistema óseo, ocular y digestivo^18^.

#### Procedimiento de retirada de los GCS: desescalada

En pacientes en los que el tratamiento con GCS ha sido efectivo o se ha iniciado un tratamiento biológico, debe procederse a su reducción de forma gradual hasta intentar completar la retirada de los GCS. De forma similar, los pacientes con respuesta no-T2 probablemente no responderán al tratamiento con GCS, y en ellos, evitar aumentar la dosis e incluso proceder a intentar su retirada de manera sistemática, y buscar otras alternativas terapéuticas como el tratamiento con azitromicina o la termoplastia^37^, ^56^, ^57^.

Aunque la GINA recomienda dosis de mantenimiento no superiores a 7,5 mg/día^7^ y, por tanto, el proceso de retirada debería ser simple, la realidad es que, en general, se usan dosis muy superiores, que de media pueden llegar a los 22 mg/día^13^. Al consultarles sobre el proceso de desescalada, los panelistas destacaron la importancia de realizar una desescalada progresiva y con el objetivo de detectar una posible IS, los pacientes que hayan recibido una dosis acumulada de GCS superior a 2 g de equivalente de prednisona en el último año, deben realizarse un estudio de cortisol plasmático a la primera hora de la mañana.

La IS secundaria se produce como resultado de cualquier proceso que interrumpa el normal funcionamiento del eje hipotalámico y cause una disminución en la secreción de hormonas de la corteza suprarrenal. La causa más común de IS secundaria es la administración de GC en dosis supra fisiológicas durante ≥ 1 mes. La IS debida a GCS no está bien conocida o diagnosticada, pero a menudo se asocia con un bienestar reducido y puede ser potencialmente mortal en caso de una crisis suprarrenal^58^.

Los panelistas consultados mostraron su acuerdo en que en el proceso de reducción/retirada de los GCS se deben tener en cuenta múltiples factores que pueden influir tanto la intensidad como la velocidad de la desescalada. Entre estos factores, acordaron que deben tenerse en cuenta en el diseño del proceso de desescalada la duración del tratamiento, la presencia y el tipo de EA, y el riesgo de presentarlos^36^, ^48^. Sin duda, y con el acuerdo de los panelistas, el EA más relevante que puede presentarse en el proceso de retirada de los GCS es la IS^18^ que, además, requiere tratamiento sustitutivo y la implementación de medidas preventivas. La IS puede ocurrir con cualquier vía de administración, duración, posología o enfermedad subyacente. Los panelistas reconocieron que el riesgo es mayor con dosis >5 mg/día de equivalente de prednisona durante > 4 semanas y que la recuperación del eje suprarrenal es impredecible, pero puede tardar varios meses, lo que requiere un régimen lento de suspensión de GCS, incluso cuando la terapia con esteroides ya no sea necesaria para el asma^59^. Este hecho condiciona que, especialmente en todos aquellos pacientes que hayan recibido una dosis acumulada de GCS superior a 2 g en el último año, deba realizarse un estudio de cortisol plasmático a primera hora de la mañana (debido al ritmo circadiano del mismo) cuando se quiera reducir la dosis de GCS por debajo de 5 mg/día^36^, ^60^, ^61^.

Las actuales guías y recomendaciones no establecen pautas para la desescalada de los GCS^7^, ^8^, ^62^. En el estudio PONENTE, un estudio de fase IIIb multicéntrico, abierto y de un solo brazo que evaluó la eficacia y seguridad de la reducción de la dosis diaria de GCS después del inicio de un tratamiento biológico con benralizumab en pacientes con asma grave^60^, ^61^, se propone un algoritmo de desescalada adaptable que los panelistas consideraron adecuado. Brevemente, cuando la dosis diaria de GCS de un paciente en tratamiento de mantenimiento supere los 20 mg/día se debe reducir la dosis 5 mg/día cada semana y si supera los 10 mg/día hasta los 20 mg/día se debe reducir la dosis en 5 mg/día cada quincena hasta llegar a la dosis diaria de 10 o 20 mg, respectivamente. A su vez, cuando la dosis diaria de GCS de un paciente en tratamiento de mantenimiento supere los 7,5 mg/día hasta los 10 mg/día se debe reducir la dosis en 2,5 mg/día cada quincena hasta llegar a la dosis diaria de 7,5 mg. Y finalmente, cuando la dosis diaria de GCS de un paciente en tratamiento de mantenimiento supere los 5 mg/día hasta los 7,5 mg/día se debe reducir la dosis en 2,5 mg/día cada mes hasta llegar a la dosis diaria de 5 mg.

### Los GCS en ciclos cortos para el tratamiento de la crisis de asma

#### Definiciones de un ciclo corto de GCS

Aunque un ciclo corto de GCS por vía oral puede ser beneficioso para mejorar los síntomas y el control del asma moderado o grave, existe evidencia de que la terapia con corticoides inhalados y agonistas β2 adrenérgicos de acción prolongada (GC inhalados [GCI]-LABA) es el tratamiento inicial adecuado para lograr el control del asma en estos pacientes y que la administración de un ciclo corto de GCS no proporciona ningún beneficio clínico significativo^63^.

El uso más frecuente de GCS en el tratamiento del asma grave se produce durante una exacerbación del asma, definida como un aumento progresivo de los síntomas suficiente para requerir un cambio de tratamiento^7^. El panel de expertos consultado estuvo de acuerdo en que, aunque el tipo de exacerbación (eosinofílica o no eosinofílica) puede influir en la respuesta al tratamiento con GCS, añadirlos al tratamiento con broncodilatadores mejora la evolución de las exacerbaciones, incluso en pacientes con asma no eosinofílica. Además, coincidieron en que el tratamiento precoz de las exacerbaciones graves del asma con GCS se considera un estándar de atención y se recomienda su administración dentro de la primera hora de la presentación^64^. En el contexto de exacerbaciones graves, este cambio de tratamiento suele requerir de un ciclo corto de GCS, que ha demostrado ser útil para evitar la necesidad de atención en el servicio de urgencias, ingresos hospitalarios y para prevenir la recaída de la exacerbación en las siguientes semanas^65^.

Esta práctica representa en la actualidad un uso apropiado de GCS en el asma. Sin embargo, al tratarse de medicamentos altamente accesibles y disponibles sin receta en algunos países, los GCS se utilizan inadecuadamente en ocasiones como medicación de rescate o se autoadministran por parte del paciente sin indicación médica.

Los panelistas consultados estuvieron de acuerdo en la imposibilidad de establecer una dosis fija de GCS para un ciclo corto, pero se pusieron de acuerdo en que un ciclo corto de GCS se puede definir como aquel que consiste en una dosis diaria fija de 50 mg de equivalente de prednisona en forma de dosis única matutina, o bien, una dosis diaria de 0,5-1 mg/kg del peso ideal por día de equivalente de prednisona, con un máximo de 40-50 mg. En cuanto a la duración, consideraron que el estándar de un ciclo corto de GCS eficaz debe estar entorno a los 5 y 7 días y no debe superar los 15 días. Notablemente, los panelistas consultados expresaron su desacuerdo acerca de la consideración de un tratamiento inferior a 30 días como un ciclo corto de GCS, revelando su interés en restringir su duración todo lo posible.

#### Indicaciones de los ciclos cortos de GCS

La eficacia de los GCS es heterogénea y, en general, se ha demostrado que la eficacia del tratamiento de GCS en ciclos cortos es mayor en el asma eosinofílica^7^. En diversos estudios que incluyeron pacientes con exacerbación asmática, EPOC o tos crónica, la ausencia de eosinofilia evidente se ha relacionado con una resistencia total o parcial al efecto del GCS^30^, aunque hay trabajos que no encuentran diferencia entre pacientes con presencia significativa o no de eosinofilia en esputo^66^. No obstante, se han asociado varios factores con la capacidad de respuesta a los GCS, pero son pocos los estudios que apliquen un enfoque integrado para identificar patrones de respuestas diferenciales y, en general, se acepta que añadir GCS a tratamientos broncodilatadores mejora (al menos parcialmente) la evolución de las exacerbaciones en pacientes con asma no eosinofílica^67^. Los panelistas consultados coincidieron en que los ciclos cortos de GCS constituyen el tratamiento específico de las exacerbaciones graves, que acelera su resolución y previene recaídas, aunque los beneficios de los GCS en ciclos cortos para el tratamiento del asma pueden depender de la dosis administrada o su duración. Por otra parte, desde la perspectiva de los panelistas consultados, no está claro que añadir un ciclo corto de GCS proporcione ningún beneficio clínico significativo en el inicio del tratamiento de mantenimiento en pacientes con asma moderada a grave.

El uso de los ciclos cortos de GCS en la práctica habitual sigue siendo, en parte, empírico. En general, su uso dura pocos días y las dosis pueden ser mucho más altas que cuando se utilizan como tratamiento de mantenimiento^7^, ^32^. Se conoce que ciclos cortos de GCS con dosis similares a las acordadas por el panel de expertos consultado en este trabajo (0,5-1 mg/kg del peso ideal por día de prednisona o equivalente hasta un máximo de 50 mg) son eficaces reduciendo las exacerbaciones en pacientes tratados en atención primaria u hospitalaria, la necesidad de atención hospitalaria y las dosis de β-agonistas de rescate^30^.

También hubo acuerdo en el panel de expertos respecto al tipo de administración: siempre que los pacientes puedan tolerar la terapia oral, no hay ningún beneficio comprobado de la administración de GCS por vía intramuscular o intravenosa^65^.

#### Seguimiento y duración

El uso de GCS en ciclos cortos es más frecuente que su uso en mantenimiento^13^, ^68^. Es importante tener en cuenta los tratamientos de otras enfermedades concomitantes que puedan requerir ciclos cortos de GCS^69^. Sin embargo, no existe un consenso claro sobre la duración de un ciclo corto de GCS para el tratamiento del asma. Algunos autores han propuesto que sería aquel que resulta inferior a 30 días^69^. Al consultar al panel de expertos sobre el seguimiento y la duración de los ciclos cortos, expresó su acuerdo en que los pacientes adultos con un plan personal de manejo del asma y tratamiento con GCI pueden reducir la duración del ciclo a 5 días, y en que los ciclos cortos inferiores a 2 semanas no precisan una retirada gradual, coincidiendo con las recomendaciones de la GINA 2020 y la GEMA 5.0^7^, ^8^, ^70^. En parte, estas recomendaciones se sustentan en una revisión sistemática que concluye que la evidencia no es robusta para sostener que ciclos más cortos o con dosis más bajas, sean menos efectivos que ciclos más largos o a mayores dosis^27^. Otros paneles de expertos internacionales coinciden en la recomendación de no sobrepasar los 7 días^36^. Sin embargo, no se ha consensuado si la dosis debe permanecer estable o si se precisa adaptación de la dosis de GCS de modo individualizado, lo que haría poco probable la aplicación sistemática de dosis «ideales»^36^. Por el contrario, en otro consenso internacional reciente, aunque apuntan que dosis mayores de 40 mg de GCS no parecen tener mayor efecto beneficioso, sí parecen asociarse a EA, por lo que no aconsejan ningún tiempo de duración del ciclo corto^48^.

Hay que recordar que la tasa de recaída en un plazo de 30 días tras una visita a urgencias u hospitalización en una exacerbación por asma alcanza el 9,2% en EE. UU. y el 4,7% en Reino Unido^23^, siendo esta cifra en España del 6,25% para un plazo menor de 15 días^71^. Estos datos, sumado a que cada año de exposición a 4 o más ciclos de GCS, actualmente o en el pasado, deriva en un riesgo 1,20 veces mayor para tener un EA en el año actual^15^, ^27^, el panel de expertos consultado estimó apropiado recomendar la evaluación del desarrollo de IS en aquellos pacientes que reciban > 4 ciclos cortos de GCS por año.

### Efectos adversos y carga acumulada de GCS

A pesar de su eficacia, la administración de GCS puede asociarse con EA graves^13^, ^14^, ^15^, ^16^, ^17^. Desafortunadamente, los datos de la literatura sobre los EA asociados al uso de GCS en asma grave son limitados y en su mayoría retrospectivos, debido a que los ensayos aleatorizados controlados se realizan durante periodos limitado y no están diseñados para estudiar los EA a largo plazo; por ello, los resultados deben interpretarse con cautela.

Al consultar con el panel de expertos sobre los EA relacionados con el uso de GCS estuvieron de acuerdo en que dependen de la dosis y son acumulativos en el tiempo cuando se trata de una terapia de mantenimiento. Además, consideraron importante consignar en la historia clínica del paciente la carga acumulada de GCS anual en cualquier tipo de tratamiento, puesto que la evidencia reciente sugiere que incluso ciclos cortos de GCS pueden asociarse con EA y cada prescripción de GCS supone una carga acumulada, independientemente de la dosis y duración^16^, ^72^. En relación con la dosis acumulada, hubo consenso entre los panelistas que no solo las dosis acumuladas de 2,5 g de prednisona o equivalente en un año aumentan significativamente el riesgo de EA, sino que la aparición de estos constituye un riesgo desde dosis acumuladas de 1,0 g de prednisona o equivalente en un año. Además, hubo acuerdo en que se puede considerar un riesgo de presentar EA con más de 4 ciclos o más de 30 días al año de tratamiento con GCS. Los EA más importantes relacionados con GCS se ilustran en la tabla 4 18, 73.

**Tabla 4:** Efectos adversos relacionados al uso de glucocorticoides

Órgano afectado/vía metabólica	Efectos adversos más frecuentes
Metabolismo: hidratos de carbono	Diabetes mellitus
Metabolismo: lípidos	Obesidad, dislipidemia
Metabolismo: proteínas	Atrofia muscular
Piel	Hirsutismo, acné, estrías
Ojo	Cataratas, exoftalmos, glaucoma
Cardiovascular	Hipertensión, aterosclerosis, Infarto de miocardio, insuficiencia cardiaca, accidente cerebrovascular
Gastrointestinal	Dispepsia, disfagia, gastritis, úlceras gástricas y duodenales, pancreatitis
Neuropsiquiátrico	Insomnio, seudotumor cerebro, alteraciones conductuales, depresión, ansiedad
Genitourinario	Amenorrea, disminución de libido
Renal	Hipopotasemia, insuficiencia renal
Hueso	Osteoporosis y fractura osteoporótica, necrosis avascular
Respiratorio	Apnea del sueño, neumonía

El seguimiento de las recomendaciones de las guías para evitar los EA a los GCS, como administrar bifosfonatos orales, calcio o vitamina D, es bajo y muchas veces referido al uso crónico de los mismos y no a los ciclos cortos^41^.

La falta de consenso sobre la duración de los ciclos cortos repercute en los EA derivados del uso de los GCS. Un estudio realizado en EE. UU. demostró que a los 30 días del inicio una prescripción de GCS por cualquier causa, aumenta significativamente el riesgo de EA^16^. En cuanto al balance entre eficacia y seguridad, el panel de expertos consultado estuvo de acuerdo en que cualquier paciente que supere un uso acumulado de > 1 g/año de equivalente de prednisona, debería ser correctamente revaluado para identificar posibles áreas de mejora en el manejo del asma.

Al consultar con panel de expertos sobre el coste sociosanitario asociado al tratamiento del asma, estuvieron de acuerdo en que el tratamiento con GCS se asocia con un aumento del gasto sanitario, en parte debido a la necesidad de resolver los EA relacionados^38^, ^74^.

Este estudio cuenta con algunas limitaciones. El hecho de que se base en las opiniones expertas de los participantes en él hace que las conclusiones resumidas a lo largo del estudio no sean equiparables a las de las guías de práctica clínica. Además, los resultados recogidos en este consenso pueden no reflejar la práctica clínica habitual de todos los profesionales sanitarios que manejan pacientes con asma puesto que el panel fue compuesto por expertos en la materia con el fin de recoger no solo su experiencia en la práctica clínica sino también sus conocimientos en la materia.

A pesar de estas limitaciones, el objetivo de este estudio fue recoger y compartir la experiencia en práctica clínica real de los participantes en el contexto de un área no totalmente cubierta por las guías de práctica clínica. En este sentido, la mayor fortaleza de este estudio consiste en la participación de 48 expertos en neumología y alergología del ámbito español que aseguran la representación de una amplia gama de conocimientos y experiencia relevantes en el manejo del paciente con asma grave. Además, el diseño anónimo, con los criterios de consenso previamente definidos y la consistente participación de los panelistas son puntos fuertes que refuerzan sus resultados.

## Conclusiones

Aunque el uso de los GCS ha sido muy extendido debido a su efectividad, su perfil de seguridad ha puesto de manifiesto la necesidad de reducir su uso en la práctica clínica habitual. El panel de expertos consultado ha puesto de manifiesto su acuerdo en la necesidad de esta reducción y ha dejado patente que esta iniciativa forma parte de su práctica clínica habitual pese a que las recomendaciones de algunas de las guías de manejo del asma más utilizadas no ajusten al mínimo el uso de dicho tratamiento. Este consenso sobre el uso de los GCS en el ámbito español puede ser de utilidad como guía en ámbitos clínicos en que no se esté ajustando al mínimo el uso de los GCS con el objetivo común de reducir al mínimo la aparición de EA asociados a dicho tratamiento.

## Contribuciones de los autores

Todos los autores contribuyeron de forma equitativa en la concepción y diseño del estudio, adquisición de datos o análisis e interpretación de los datos, en la escritura del borrador del artículo o revisión crítica del contenido intelectual y en la aprobación definitiva de la versión que se presenta.

## Financiación

Este proyecto ha sido promovido y financiado por la SEPAR mediante una beca sin restricciones facilitada por la compañía AstraZeneca España.

## Conflicto de intereses

JD-O ha recibido honorarios como ponente, asesor científico o participante en estudios clínicos: AstraZeneca, BIAL, Chiesi, GlaxoSmithKline, LETI-Pharma, Novartis, Sanofi, Teva. JDR ha recibido honorarios como ponente, asesor científico o participante de estudios clínicos de: AstraZeneca, Bial, Chiesi, Faes, GlaxoSmithKline, Novartis, Sanofi, Teva. XMG ha recibido honorarios como ponente, asesor científico o participante de estudios clínicos de: AstraZeneca, Boehringer Ingelheim, Chiesi, Faes, GlaxoSmithKline, Menarini, Mundifarma, Novartis, Sanofi, Teva. MB-A ha recibido honorarios como ponente, asesor científico o participante de estudios clínicos: AstraZeneca, Chiesi, GlaxoSmithKline, Menarini, Novartis, Sanofi, Teva.
