# Supplementary material for: Use of systemic glucocorticoids for the treatment of severe asthma: Spanish Multidisciplinary Consensus
Source: Open Respir Arch. 2022 Sep 6;4(4):100202. [Article in Spanish] doi: 10.1016/j.opresp.2022.100202 (PMC10369533; doi:10.1016/j.opresp.2022.100202)
Supplement: Supplementary file 1 [file mmc1.doc]

Uso de glucocorticoides sistémicos para el tratamiento del asma grave: Consenso multidisciplinar español

# TABLA SUPLEMENTARIA

Tabla S 1. Panel de expertos consultados en este trabajo

| **NOMBRE** | **FILIACIÓN** |
| --- | --- |
| Alberto Luna Porta | Hospital Universitario La Paz, Madrid |
| Alfredo de Diego Damiá | Hospital Universitario la Fe, Valencia |
| Alicia Habernau | Hospital de Mérida, Badajoz |
| Alicia Padilla Galo | Hospital Regional Universitario de Málaga |
| Ana Gomez-Bastero | Hospital Virgen Macarena, Sevilla |
| Ana Isabel Sogo Sagardía | Hospital de Sabadell. Corporació Parc Taulí, Barcelona |
| Ana Navarro | Hospital el Tomillar, Sevilla |
| Andrea Trisán | Hospital Universitario Puerta de Hierro-Majadahonda, Madrid |
| Angel Ferrer | Hospital Vega Baja, Alicante |
| Antolín Lopez Viña | Hospital Universitario Puerta de Hierro-Majadahonda, Madrid |
| Auxiliadora Romero Falcón | Hospital Virgen del Rocío, Sevilla |
| Carlos Almonacid Sánchez | Complejo Hospitalario Universitario de Toledo (CHUT) |
| Carlos Colás | Hospital Clínico, Zaragoza |
| Carlos Martínez Rivera | Hospital Universitari Germans Trias i Pujol, Barcelona |
| Carlos Melero Moreno | Hospital Universitario 12 de Octubre, Madrid |
| Carolina Cisneros Serrano | Hospital Universitario de La Princesa, Madrid |
| Darío Antolín | Hospital Ramón y Cajal, Madrid |
| Elisa Gómez Torrijos | Hospital de Ciudad Real |
| Francisco Casas Maldonado | Complejo Hospitalario Universitario de Granada, Granada |
| Francisco Javier González Barcala | Hospital Clínico Universitario de Santiago de Compostela, A Coruña |
| Gregorio Soto Campos | Hospital de Jerez, Cádiz |
| Ignacio Antépara | Hospital Universitario de Basurto, Bizkaia |
| Irina bobolea | Hospital Clínic, Barcelona |
| Isabel Urrutia Landa | Hospital de Galdakao, Bizkaia |
| Ismael García Moguel | Hospital 12 de Octubre, Madrid |
| José Ángel Carretero Gracia | Hospital Royo Villanova, Zaragoza |
| Jose Antonio Castilo Vizuete | Hospital Universitari Dexeus, Barcelona |
| José María Olaguibel | Complejo Hospitalario de Navarra |
| José María Vega Chicote | Hospital Regional Universitario de Málaga |
| Juan Carlos Miralles | Hospital Reina Sofía, Murcia |
| Lorena Soto | Hospital de la Santa Creu i Sant Pau, Barcelona |
| Luis Alonso González Sánchez | Hospital General La Mancha-Centro, Ciudad Real |
| Luis Pérez de Llano | Hospital Lucus Agusti, Lugo |
| Magdalena Lluch Bernal | Hospital Universitario La Paz, Madrid |
| Mar Fernández Nieto | Fundación Jiménez Díaz, Madrid |
| Mar Gandolfo Cano | Hospital Universitario Fuenlabrada, Madrid |
| Mar Mosteiro Añón | Hospital Álvaro Cunquiero, Pontevedra |
| María Vázquez de la Torre Gaspar | Hospital Infanta Leonor, Madrid |
| Mercedes Rodríguez | Hospital Universitario de Alcalá de Henares, Madrid |
| Miguel Perpiñá Tordera | Hospital universitario y politécnico la fe, valencia |
| Pilar Cebollero Rivas | Complejo Hospitalario de Navarra |
| Remedios Cárdenas | Hospital Universitario de Guadalajara, Madrid |
| Rocio García García | Hospital 12 de Octubre, Madrid |
| Santiago Quirce | Hospital Universitario La Paz, Madrid |
| Valentina Gutiérrez | Hospital Universitario Dr. Peset, Valencia |
| Vicente Plaza Moral | Hospital de la Santa Creu i Sant Pau, Barcelona |
| Victoria García Gallardo | Hospital Universitario Río Hortega, Valladolid |
| Virginia Bellido | Hospital Universitario Virgen Macarena, Sevilla |
